# An Automated Clinical Alert System for Newly-Diagnosed Atrial Fibrillation

**DOI:** 10.1371/journal.pone.0122153

**Published:** 2015-04-07

**Authors:** David A. Cook, Felicity Enders, Pedro J. Caraballo, Rick A. Nishimura, Farrell J. Lloyd

**Affiliations:** 1 Knowledge Delivery Center, Mayo Clinic, Rochester, MN, United States of America; 2 Division of General Internal Medicine, Mayo Clinic College of Medicine, Rochester, MN, United States of America; 3 Mayo Clinic Online Learning, Mayo Clinic College of Medicine, Rochester, MN, United States of America; 4 Department of Health Sciences Research, Mayo Clinic College of Medicine, Rochester, MN, United States of America; 5 Division of Cardiovascular Diseases, Mayo Clinic College of Medicine, Rochester, MN, United States of America; 6 Division of Hospital Medicine, Mayo Clinic College of Medicine, Rochester, MN, United States of America; Boston University School of Medicine, UNITED STATES

## Abstract

**Objective:**

Clinical decision support systems that notify providers of abnormal test results have produced mixed results. We sought to develop, implement, and evaluate the impact of a computer-based clinical alert system intended to improve atrial fibrillation stroke prophylaxis, and identify reasons providers do not implement a guideline-concordant response.

**Materials and Methods:**

We conducted a cohort study with historical controls among patients at a tertiary care hospital. We developed a decision rule to identify newly-diagnosed atrial fibrillation, automatically notify providers, and direct them to online evidence-based management guidelines. We tracked all notifications from December 2009 to February 2010 (notification period) and applied the same decision rule to all patients from December 2008 to February 2009 (control period). Primary outcomes were accuracy of notification (confirmed through chart review) and prescription of warfarin within 30 days.

**Results:**

During the notification period 604 notifications were triggered, of which 268 (44%) were confirmed as newly-diagnosed atrial fibrillation. The notifications not confirmed as newly-diagnosed involved patients with no recent electrocardiogram at our institution. Thirty-four of 125 high-risk patients (27%) received warfarin in the notification period, compared with 34 of 94 (36%) in the control period (odds ratio, 0.66 [95% CI, 0.37–1.17]; p = 0.16). Common reasons to not prescribe warfarin (identified from chart review of 151 patients) included upcoming surgical procedure, choice to use aspirin, and discrepancy between clinical notes and the medication record.

**Conclusions:**

An automated system to identify newly-diagnosed atrial fibrillation, notify providers, and encourage access to management guidelines did not change provider behaviors.

## INTRODUCTION

Ongoing advances in clinical medicine create new opportunities for patient-centered, high-value care, but achieving this potential will require new models for translating evidence into practice. One such approach uses the electronic medical record (EMR) to proactively notify providers of opportunities to optimize care.[1–5] Such clinical alerts have been used to identify potential drug-drug interactions or dose adjustments when prescribing[1] and to notify providers of critical test results[4, 6] or unexpected changes in clinical status.[3] Such interventions have had mixed effects because clinicians often ignore or override the alert[7, 8] or fail to act appropriately;[9] or because of unintended negative consequences such as increased use of follow-up tests without clinical benefit.[10]

A minority of alerts often accounts for the majority of clinically significant benefits, suggesting that focusing on such high-yield alerts (and suppressing low-yield alerts) may enhance the overall value of the alert system.[1, 11] Despite efforts to better understand the factors that influence successful test notification,[1, 11, 12] much remains to be learned about the successful implementation of this technology.[13, 14] One possible solution is to merge the novel finding (e.g., test abnormality) with other clinical information in the EMR to present a more customized notification, which should hypothetically reduce alert fatigue[14] and increase the likelihood of a clinically appropriate response. This study evaluated the effectiveness of such a tailored clinical alert for atrial fibrillation.

Atrial fibrillation is a common clinical problem and is associated with an increased incidence of stroke.[15] Anticoagulation with warfarin significantly reduces stroke incidence,[16] and the benefit is greatest in those with higher risk of stroke. To help clinicians make decisions about anticoagulation, evidence-based guidelines have been developed along with simple rules that classify the patient's risk profile. The "CHADS2" score[17] uses a patient's history of Congestive heart failure, Hypertension, Age>75, Diabetes, and Stroke to create a risk profile; those with a CHADS2 score >2 should receive anticoagulation with warfarin unless clinically contraindicated.[18, 19] Unfortunately, anticoagulation rates remain relatively low,[20–24] meaning that many patients remain at unnecessarily higher risk of stroke.[23, 25] Automated notifications to providers to identify patients with newly-diagnosed atrial fibrillation might help address this gap in quality care. Aside from one clinical trial in progress,[26] we are not aware of any studies of automated result notification of cardiac rhythm disturbances detected on an electrocardiogram (ECG).

The purpose of the present study was to develop, implement, and evaluate the impact of an automated clinical alert system for atrial fibrillation and identify reasons providers do not implement a guideline-concordant response. The intended goal of the clinical alert system was to increase the rate of appropriate prescription of warfarin for stroke prevention.

## METHODS

### Overview

We conducted a cohort study using historical controls, comparing the prescribing patterns of providers in the first 3 months of the clinical alert against a historical control (the corresponding 3-month period the previous year).

### Notification system and clinical rule

MayoExpert is a multifaceted computer-based knowledge resource integrated with the EMR across a multi-site health system.[27] MayoExpert includes answers to frequently-asked questions, institution-approved care process pathways, a directory of topic-specific experts, a portfolio for licensure and credentialing, and integration with a system to notify providers of urgent or unexpected test results. The test notification system was initially developed for two cardiac dysrhythmias diagnosed using the surface ECG: newly-diagnosed atrial fibrillation not on anticoagulation (the focus of the present study) and prolonged QT interval.[28]

In collaboration with board-certified cardiologists we developed a decision rule defining an atrial fibrillation event warranting notification (i.e., previously undiagnosed and not already taking warfarin). In the final rule, a notification would be triggered if all of the following were true:

1. Atrial fibrillation is present on the final ECG interpretation;
2. Atrial fibrillation is not listed on the patient’s EMR problem list;
3. Atrial fibrillation is not present on any ECG in the past five years;
4. Recent (within the past 2 months) international normalized ratio (INR) <2, or no recent INR available, or current heart rate >100;
5. Not hospitalized on a cardiology or cardiac surgery service.

All ECG interpretations are suggested by the ECG analysis system (MUSE, GE Heathcare), reviewed by an ECG technician, and confirmed by a cardiologist before reaching the EMR as final results.

The decision rule was implemented in the EMR at the Mayo Clinic in Rochester, MN. At the time of this study, Mayo used Centricity Enterprise (GE Healthcare, Seattle, WA) and its integrated expert rule engine (Blaze Advisor, Fair Isaac Corporation, Minneapolis, MN) as the main EMR infrastructure. The decision rule was developed by a multidisciplinary team including clinicians, informaticians, and programmers. Prior to actual use in clinical practice the rule was iteratively pilot tested and revised by "silent" operation, in which the system notified the development team (rather than the actual provider), and patient records were reviewed to confirm or disconfirm the notification as accurate. The notification system became operational in practice on April 30, 2009.

**Fig 1:**
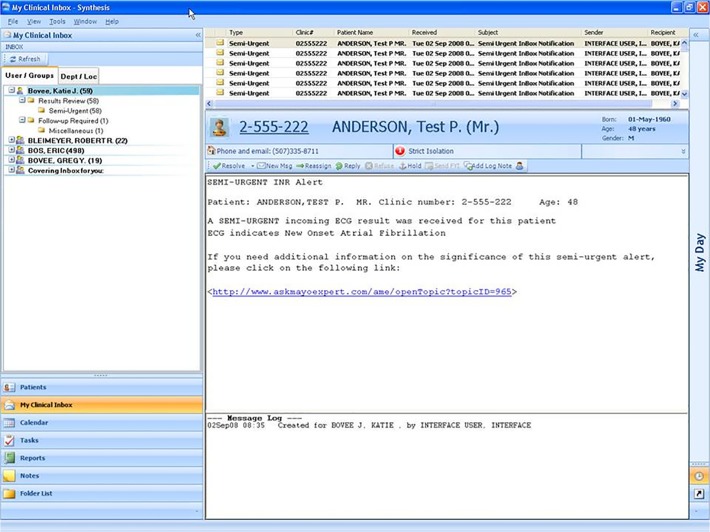
Screen shot of the EMR notification message. Note that the "patient" in this screen shot is not a real person. The link would take them directly to topic-specific information in the MayoExpert knowledge delivery system (see Fig 2).

**Fig 2:**
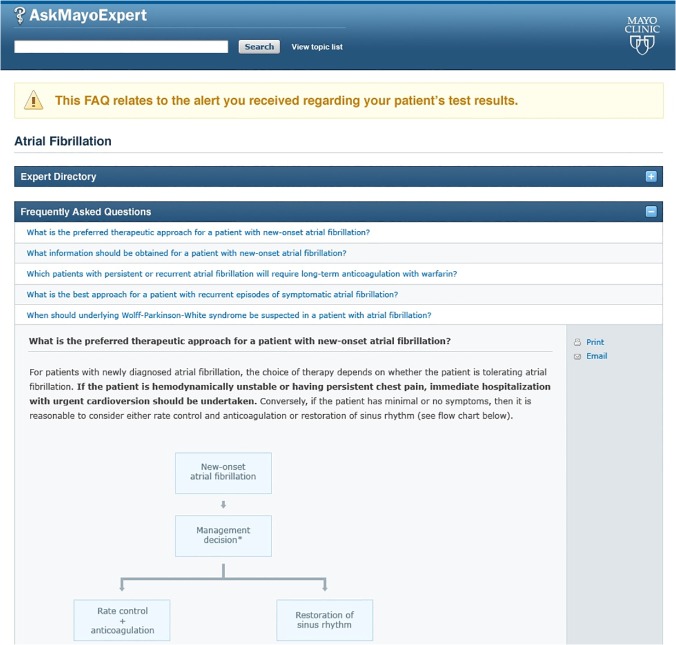
Screen shot of topic-specific information in the MayoExpert knowledge delivery system. The link in the EMR message would take providers to this screen.

Notifications appear in the recipient’s EMR messaging system Inbox with a priority marker indicating the need for prompt attention (Fig 1). The message notified the physician that, "A semi-urgent incoming ECG result was received for this patient. ECG indicates new onset atrial fibrillation. If you need additional information on the significance of this semi-urgent alert, please click on the following link." This link would open a screen in MayoExpert[27] (Fig 2) containing additional information on atrial fibrillation including the indications for stroke prophylaxis.

### Participants

Although the notification system monitors patients and alerts providers for both hospitalized and ambulatory patients, we excluded ambulatory patients for pragmatic reasons (the longer time delay before first follow-up, and the use of paper prescribing which limited accurate abstraction of medication changes). We also excluded patients on a cardiology service because these services have independent processes for managing abnormal ECGs. For all notifications triggered in a non-cardiology hospitalized patient in the period December 2009 to February 2010 we extracted clinical information as noted below. We created a historical comparison cohort by identifying patients with newly-diagnosed atrial fibrillation in the corresponding 3-month period one year prior (December 2008—February 2009). To identify this comparison group, we retrospectively applied the decision rule above to all ECGs showing atrial fibrillation during that time period, thus identifying those patients for whom a notification would have been triggered. We selected this time frame to control for possible seasonal variation in patient populations.

### Ethics statement

This study was approved by the Mayo Clinic Institutional Review Board. Although it was neither feasible nor required by the review board that we obtain content from individual patients, Mayo Clinic offers patients the opportunity to opt out of all studies using their records, and we adhered to this policy. Information was extracted from medical records using patient unique identifiers without recording names. All data were analyzed anonymously.

### Outcomes and instruments

We identified two primary outcome measures—a process measure and a physician behavior. The primary process measure was the number and accuracy of notifications delivered. We used coder-extracted information from the EMR (see below) to verify the accuracy of notification (i.e., was atrial fibrillation newly-diagnosed?). The primary behavior measure was the number of high-risk patients with newly-diagnosed atrial fibrillation eligible for warfarin anticoagulation who received a prescription for warfarin within 30 days of diagnosis. We defined a warfarin-eligible patient as one with no active bleeding and no use of warfarin at hospital admission. We defined a high-risk patient as one with CHADS2≥2, a risk level at which warfarin anticoagulation is recommended by guidelines[18, 19] except in the case of active bleeding. Warfarin was counted as prescribed if warfarin was listed as a current medication in the EMR medication registry, or if warfarin was listed as a medication on the hospital dismissal summary.

Secondary outcomes were use of an appropriate medication (warfarin for any warfarin-eligible patient or aspirin for patients at low risk of stroke [CHADS2<2]), the time delay before prescribing warfarin in warfarin-eligible patients, and the reason for not prescribing warfarin among high-risk warfarin-eligible patients who did not receive a prescription.

Two research assistants experienced in chart abstraction independently reviewed the medical record using a detailed form to determine whether the patient had a prior history of atrial fibrillation and to extract demographic data (emphasizing information to calculate the CHADS2 score [history of congestive heart failure, hypertension, diabetes, and stroke]) and medications prior to hospitalization. Each coder was blinded to the others' codes, but it was impossible to blind coders to the date range of hospitalization. However, to minimize potential bias one coder was kept unaware of the study intent. All discrepancies were resolved by consensus. One author (FJL) reviewed the charts of all patients with newly-diagnosed atrial fibrillation and CHADS2 ≥2 (in both study periods) for whom warfarin was not prescribed to determine the documented reason for not prescribing warfarin.

### Statistical analysis

We used the chi-squared test to compare the proportions of patients prescribed indicated medications in the notification and control periods. We used logistic regression to repeat these comparisons while controlling for CHADS2 score and age. We used time-to-event analyses with Cox proportional hazards to compare the time to warfarin prescription between time periods. Other results are reported using measures of central tendency. We used SAS 9.1.3 for all analyses, with a two-sided alpha level of 0.05.

## RESULTS

### Number and accuracy of notifications

In the three months following implementation of the notification system (December 2009 to February 2010) there were 16,755 ECGs performed on hospitalized patients. During this period the decision rule identified 604 patients as having potentially newly-diagnosed atrial fibrillation (3.6% of all ECGs) and notified the ordering provider via the EMR messaging system. Of these 604, 268 (44%) were ultimately judged in manual review of clinical documentation to be truly newly-diagnosed. The other (not newly-diagnosed) notifications occurred in patients without an ECG showing atrial fibrillation at our medical center in the past five years, but who had been diagnosed with atrial fibrillation at another medical center or whose last ECG-documented episode of atrial fibrillation was more than five years prior.

### Comparability of comparison group

In the control time period (before notification) we manually reviewed records and confirmed 226 patients with newly-diagnosed atrial fibrillation (from 17,520 ECGs performed on hospitalized patients). Patient demographics for both notification and control groups are shown in Table 1. Patient groups were similar except for history of congestive heart failure and diabetes, both more common in the control period. Despite this, CHADS2 scores were similar in both groups (mean [SD] 1.59 [1.19] in the notification group vs 1.56 [1.17] in the control group; p = 0.80).

**Table 1:** Demographic information for patients with newly-diagnosed atrial fibrillation.

Demographic feature	Control period No. (%), N = 226	Notification period No. (%), N = 268
Congestive heart failure*	31 (14)	18 (7)
Hypertension	146 (65)	179 (67)
Age > 75	96 (42)	109 (41)
Diabetes*	50 (22)	13 (5)
Stroke	15 (7)	22 (8)
High risk of stroke (CHADS2≥2)	113 (50)	139 (52)
Active bleeding during hospitalization	11 (5)	9 (3)
Using warfarin at admission	19 (8)	16 (6)
Using aspirin at admission	113 (50)	156 (58)
Warfarin-eligible^†^	196 (87)	244 (91)
Warfarin-eligible high-risk^†^	94 (42)	125 (47)

* p<0.05

^†^ Warfarin-eligible = no active bleeding and no use of warfarin at admission.

Thirty-five patients were using warfarin at the time of hospital admission and 20 had clinically significant bleeding making them ineligible for anticoagulation (54 patients total); we did not include these patients in subsequent analyses.

### Rate and time-to-prescription of anticoagulation

We evaluated the clinical impact of the notification system by comparing the frequency of new warfarin prescription among high-risk warfarin-eligible patients (CHADS2≥2 and no active bleeding) with newly-diagnosed atrial fibrillation in the notification and control period. Among 125 high-risk warfarin-eligible patients in the notification group, 34 (27%) received a prescription for warfarin within 30 days of diagnosis, compared with 34 of 94 (36%) in the control group (odds ratio 0.66 [95% CI, 0.37–1.17]; p = 0.16). We repeated this comparison among all warfarin-eligible patients (both high and low risk of stroke) while controlling for CHADS2 score and age; these analyses again showed no difference between groups (adjusted odds ratio 0.91 [95% CI, 0.60–1.38]; p = 0.65).

**Fig 3:**
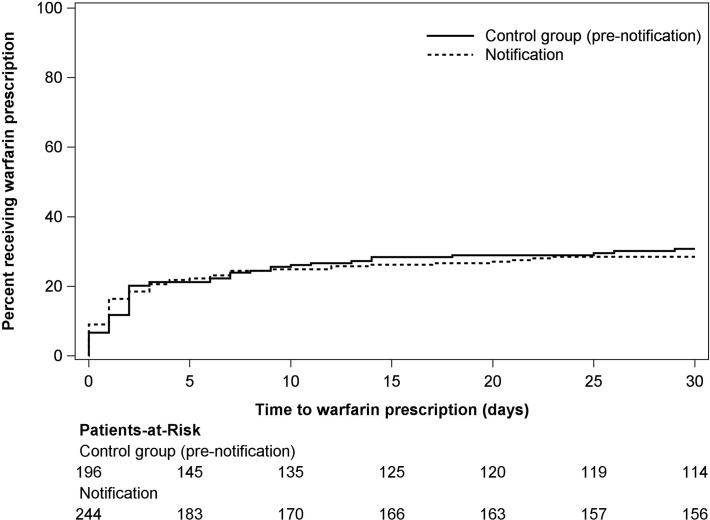
Time to prescription of warfarin following new diagnosis of atrial fibrillation. This figure depicts the cumulative percentage of warfarin-eligible patients (N = 440) who received a prescription for warfarin in the first 30 days following the initial ECG showing atrial fibrillation. The difference between groups was not statistically significant (p = 0.74).

We evaluated the time to warfarin prescription using a time-to-event analysis. Among all warfarin-eligible patients (N = 440) the time to warfarin prescription was virtually identical in the notification and control periods (Fig 3; p = 0.74).

As a planned secondary outcome, we compared the frequency of an appropriate medication prescription (warfarin for any warfarin-eligible patient, or aspirin for warfarin-eligible low-risk patients [CHADS2<2]). An appropriate medication was prescribed for 109 of 244 (45%) warfarin-eligible patients in the notification period, compared with 85 of 196 (43%) in the control period (odds ratio 1.05 [95% CI, 0.72–1.54]; p = 0.78). Adjusting this analysis for CHADS2 score and age revealed similar results (adjusted odds ratio 1.12 [95% CI, 0.76–1.66]; p = 0.57).

### Reasons for non-use of warfarin

To better understand the reasons why warfarin was not prescribed, we reviewed the charts of the 151 high-risk warfarin-eligible patients with newly-diagnosed atrial fibrillation who were not prescribed warfarin (60 in the pre-notification period, and 91 during the notification period). The most common reasons documented by the hospital team are summarized in Table 2. The modal reason, documented in 34 of 151 cases, was a planned surgical procedure, followed by a choice to use aspirin instead of warfarin (N = 27). No reason was documented in 18 cases, and in 16 cases the clinical team documented that the patient was (or should be) receiving warfarin while the EMR medication record did not. Other reasons commonly cited include frequent falls, absence of atrial fibrillation on a subsequent ECG, and high risk of bleeding (see Table 2).

**Table 2:** Reasons for non-use of warfarin in patients with newly-diagnosed atrial fibrillation and high risk of stroke.

Reason*	Pre-notification period No. (%), N = 60	Notification period No. (%), N = 91
Surgical intervention planned	13 (22)	21 (23)
Choice to use aspirin	10 (17)	17 (19)
No documented reason	3 (5)	15 (16)
Clinical documentation of current warfarin use (i.e., discrepancy between medication record and clinical notes)	6 (10)	10 (11)
Frequent falls	8 (13)	6 (7)
Sinus rhythm on subsequent ECG	8 (13)	6 (7)
Patient in hospice care	5 (8)	5 (5)
Patient death	4 (7)	5 (5)
High bleeding risk not otherwise specified	5 (8)	3 (3)
Use of other anticoagulation (e.g., IV heparin)	1 (2)	6 (7)
History of gastrointestinal bleeding	1 (2)	5 (5)
Overall complexity	1 (2)	5 (5)
Patient refused	5 (8)	1 (1)
Documented CHADS2<2 (i.e., different calculation by care team)	2 (3)	3 (3)
History of stroke or intracranial bleed	0 (0)	4 (4)
Deferred to primary care provider	2 (3)	1 (1)
Intracranial appliance	1 (2)	0 (0)

* Reasons reflect clinical documentation of rationale according to care team. Numbers sum to >100% because a given patient could have more than one reason for non-use.

## DISCUSSION

We developed, implemented, and evaluated a system to alert providers of newly-diagnosed atrial fibrillation, with the intent of improving guideline-concordant use of warfarin for stroke prophylaxis. This system correctly notified providers of 268 patients with newly-diagnosed atrial fibrillation, yet we found no statistically significant difference from historical controls in the rate of warfarin anticoagulation or the time to prescribing warfarin. Reasons for not using anticoagulation after notification were not always documented, but most often noted temporary withholding of warfarin for a specific procedure, choice to use aspirin instead, and high risk of bleeding.

### Limitations and strengths

This study has several limitations. The use of historical rather than randomized controls allows the possibility of systematic differences between the study groups, although we tried to control for seasonal variation and groups were similar in most measured characteristics. We are not aware of any changes in institution procedures that would significantly influence our findings. We excluded ambulatory patients for a number of pragmatic reasons (see Methods), such that these results may not apply to non-hospitalized patients. Only one investigator reviewed charts to identify reasons that warfarin was not prescribed. We did not review all ECGs and patient charts to identify missed cases of newly-diagnoses atrial fibrillation, but during pilot testing we found the decision rule algorithm to be accurate. We did not abstract information on reason for hospitalization. There are also several strengths, including a large sample size, a control group, combined computer and human interpretations of ECGs, analyses stratifying by and adjusting for patient risk, and additional chart abstraction looking for reasons for guideline non-adherence.

### Integration with prior work

The computer decision rule notified providers of 268 patients with newly-diagnosed atrial fibrillation after sifting through over 16,000 ECGs and sending 604 notifications. Although notifications proved unnecessary (not newly-diagnosed) in about half the cases, this accuracy is much higher than that of clinical alerts for potential drug-drug interactions[8] and similar to that of systems to notify providers of changes in clinical status.[3, 29] Overall, this suggests relatively acceptable accuracy for this decision rule.

Yet provider responses to notifications were less favorable than we had hoped. Potential explanations for this discrepancy include inappropriate timing or suboptimal design of the notification message (e.g., requiring providers to click to access information); providers' failure to acknowledge the notification (e.g. alert fatigue[14]) or recognize its importance (e.g., failure to recognize a knowledge gap[30] or disagreement with recommendations[5]); and shortcomings in the knowledge resource (e.g., inefficient or non-intuitive organization[31]). These and other factors have been described as "grand challenges" to the implementation and clinical impact of computer-based clinical decision support.[32] Prior research on computer-based decision supports suggests that they often do not work as expected.[33–35] Predictors of success, which remain incompletely clarified, include integration into the clinician's workflow, provision of specific recommendations, inclusion of patient education, and requiring clinicians to justify non-adherence to recommendations.[33, 36, 37] Considering clinical alerts specifically, evidence suggests additional influential factors including providers' awareness of the severity and probability of the potential adverse event, the patient's clinical status and specific risk factors, and the strength of the evidence supporting the recommendation.[5, 11–13] A final challenge is that clinical practice is constantly changing, as illustrated by an updated risk scoring system (the CHA2DS2-VASc) that was first described[38] just as our study ended, and will require a change to our notification decision rule.

Warfarin usage in our sample (about 60% of high-risk patients, not counting those who died or who had an upcoming surgical procedure) was similar to that described in the literature (typically about 40 to 60% of eligible patients).[20, 22–25, 39] In prior studies[25, 39, 40] and in ours, many of the reasons documented for not using warfarin are inappropriate, suggesting knowledge gaps among faculty members regarding the safety of warfarin (e.g., among older patients and those at risk of falls or bleeding[41]). Such gaps between evidence and practice highlight the imperative to change provider behaviors through both notification and education.

An ongoing clinical trial is evaluating a system similar to ours for notifying providers of new-onset atrial fibrillation.[26] Other evaluations of computer-based supports to enhance atrial fibrillation management are limited. One study piloted a computer scoring algorithm to calculate risk scores.[42] Others have used computer-based tools to increase warfarin use among patients in a nursing home,[43] to optimize the timing of follow-up,[44] and to inform provider-patient treatment discussions.[45]

## IMPLICATIONS AND CONCLUSIONS

This study highlights the imperative to better understand how to translate a clinical notification into provider action. Since providers are bombarded daily with numerous alerts[4] it may help to further improve the decision rule accuracy, and to tailor the message to more clearly indicate the recommended action. To decrease unnecessary alerts, we are changing the decision rule to exclude patients already recognized as having atrial fibrillation (and who thus do not warrant an alert) by improving linkage with the EMR problem list and medication list. We also plan to adjust the notification message if the duration of atrial fibrillation is unknown (i.e., no prior ECGs available). Automated estimation of stroke risk would further help to tailor notifications.[42] It may reduce alert fatigue to use separate notifications for patients requiring anticoagulation and those with rapid ventricular response. Finally, given the rising use of anticoagulants not monitored with INR, the role of the INR in the decision rule will be revisited.

Yet the accuracy of our system was already relatively good, which highlights the critical role of human factors in translating information into action.[46] Providers now have access to virtually limitless information at the point of care, but continue to struggle in recognizing knowledge gaps, identifying practice updates, and acting on current evidence. In addition to transforming information to actionable knowledge (knowledge creation),[47] effective knowledge translation requires delivery to the right provider at the right time using a format that promotes both immediate action and knowledge retention.[48–50] Computer point-of-care knowledge resources can potentially help with both.[31, 33, 51] The science of knowledge translation is still developing,[52] but preliminary evidence suggests that favorable factors include optimal integration into clinical workflow, provision of specific recommendations, and highlighting the clinical impact of these recommendations along with the strength of the evidence.[13, 33, 36, 37] Notification via an EMR message did not appear to be an effective workflow integration in our study. Further research to iteratively test and optimize clinical alerts is needed, looking first at the impact on provider behaviors and then ensuring that behaviors translate to desired changes in clinical practice.
